# ASPM Promotes the Progression of Anaplastic Thyroid Carcinomas by Regulating the Wnt/*β*-Catenin Signaling Pathway

**DOI:** 10.1155/2022/5316102

**Published:** 2022-03-27

**Authors:** Liang Jiang, Shuai Zhang, Ning An, Guoqing Chai, Changhong Ye

**Affiliations:** ^1^Department of Head and Neck Surgery, Hubei Cancer Hospital, Tongji Medical College, Huazhong University of Science and Technology, Wuhan, China; ^2^Department of General Surgery, The Traditional Chinese Medicine Hospital of Jiangxia District, Wuhan, China; ^3^Department of Gynecology, Maternal and Child Health Hospital of Hubei Province, Tongji Medical College, Huazhong University of Science and Technology, Wuhan, China

## Abstract

**Background:**

Abnormal spindle-like microcephaly-associated protein (ASPM) is closely correlated with several malignant tumors, whereas little is known about the role of ASPM in anaplastic thyroid cancer (ATC). Herein, we sought to investigate whether ASPM is involved in the pathogenesis of ATC and the underlying mechanisms.

**Methods:**

The data from two data sets (GSE76039 and GSE33630) were extracted and analyzed for the expression of ASPM, followed by a further validation in collected ATC patients using quantitative real-time polymerase chain reaction (qRT-PCR) and western blotting. The effect of ASPM on cell proliferation, migration, invasion, and cell cycle was explored in ATC cell lines by in vitro inhibition of ASPM, while ASPM-mediated tumorigenicity was investigated in a xenograft tumor model. The involvement of Wnt/*β*-catenin signaling pathway was also investigated.

**Results:**

ASPM was overexpressed in ATC patients and cell lines. In vitro knockdown of ASPM inhibited the proliferation, migration, and invasion capabilities of ATC cells and induced cell cycle arrest. Wnt/*β*-catenin signaling was suppressed in response to ASPM inhibition, while rescue of *β*-catenin expression restored the impaired biological functions of ATC cells. In vivo transplantation of ASPM-knockdown cells inhibited the growth of tumors.

**Conclusions:**

Upregulation of ASPM promotes the malignant properties of ATC cells and contributes to tumorigenesis through the Wnt/*β*-catenin signaling pathway.

## 1. Introduction

As the most prevalent endocrine malignant disease, the incidence of thyroid cancer has been increasing rapidly during the past decades [1]. Despite that anaplastic thyroid cancer (ATC) accounts for only 1–3% of all types of thyroid cancer, it is the most lethal one with the general survival time of 3–6 months after initial diagnosis [2]. ATC can arise de novo or from existing differentiated thyroid cancers [3]. The current therapeutic approaches for thyroid cancer, including chemotherapy, radiotherapy, and surgery, only achieve suboptimal success in ATC treatment, forcing ATC patients to depend on palliative therapies [4]. Thereby, it is essential to understand the carcinogenesis of ATC and develop novel clinical treatment targets.

Abnormal spindle-like microcephaly-associated protein (ASPM) was first found to be a centrosome protein, playing a role in the regulation of neurogenesis and brain size [5,6]. Several studies have demonstrated that ASPM can also regulate G1 restriction and microtubule disassembly at spindle poles [7,8]. Besides, ASPM has been shown to be a positive regulator of the canonical Wnt signaling pathway in previous studies [9,10]. Recent work has identified that ASPM interacts with disheveled-3 (Dvl-3), a cardinal upstream regulator of canonical Wnt signaling, and inhibits its proteasome-dependent degradation, thereby increasing its protein stability and enabling *β*-catenin transcriptional activity in prostate cancer cells [11]. ASPM also promotes Wnt pathway activity and cancer stemness by positively regulating Dvl-2 and *β*-catenin in pancreatic ductal adenocarcinoma [10]. Additionally, ASPM regulates Wnt/*β*-catenin activity to facilitate neurogenesis in brain development [12]. Recently, emerging evidence has illustrated that ASPM is expressed in malignant tissues and associated with the progression of several tumors, including lung carcinoma, gastric cancer, hepatocellular carcinoma, prostate cancer, and pancreatic ductal adenocarcinoma [10,11,13–16]. However, the involvement of ASPM in ATC has not been clarified.

Herein, to address the role of ASPM in the tumorigenesis of ATC, we identified significantly upregulated ASPM expression in ATC tissues via bioinformatics analysis. The tumorigenic role of ASPM in ATC and the underlying mechanisms were also investigated in vitro and in vivo. Thus, ASPM could present as a promising therapeutic target for ATC.

## 2. Materials and Methods

### 2.1. Bioinformatics Analysis

The mRNA expression profiles of thyroid cancer tissues were extracted from two microarray data sets GSE76039 and GSE33630, which were downloaded from the Gene Expression Omnibus (GEO) database (http://www.ncbi.nlm.nih.gov/geo). GSE76039 contains 17 poorly differentiated thyroid cancer (PDTC) and 20 ATC tissues, while GSE33630 contains 11 ATC, 49 papillary thyroid cancer (PTC), and 45 normal tissues. The mRNA expression of ASPM was compared based on the extracted data.

### 2.2. Patients and Cells

Thirty pairs of anaplastic thyroid cancer (ATC) tissues and adjacent noncancerous tissues and papillary thyroid cancer (PTC) (*n* = 30 in each category) tissues were collected from patients who underwent surgical resection in Hubei Cancer Hospital. All samples were stored in liquid nitrogen immediately upon removal. This study was approved by the Ethics Committee of Hubei Cancer Hospital, and informed consent was obtained from all patients. All human studies were performed in conformity to the Declaration of Helsinki.

Human ATC cell lines (SW1736, 8505C, TTA1, C643, and ACT-1), PTC cell lines (BCPAP and KTC-1), and the normal thyroid epithelial cell line Nthy-ori 3-1 were purchased from BeNa Culture Collection (Beijing, China). The cells were incubated in DMEM or RPMI-1640 medium supplemented with 10% fetal bovine serum (FBS) (Gibco, MD, USA), streptomycin (100  U/ml), and penicillin sodium (100 U/ml) at 37°C with 5% CO_2_.

### 2.3. Quantitative Real-Time Polymerase Chain Reaction (qRT-PCR)

Total RNA was extracted from cells and tissues by TRIzol reagent (Invitrogen, USA), followed by reverse transcription into cDNA with HiScript® II Q RT SuperMix (Vazyme, China). Subsequently, qRT-PCR was performed using AceQ® Universal SYBR qPCR Master Mix (Vazyme) in a LightCycler® 480 PCR system (Roche, Switzerland). The relative mRNA expression of ASPM was normalized against GAPDH. The primers used were listed as follows: ASPM 5′-GGGAAAGGCAAATGGAAAAC-3' and 5′-CCCAAGGCCATACAAGTGTT-3'; GAPDH 5'-TCTGACGTGCCGCCTGGAGA-3′ and 5′-CAGCCCCGGCATCGAAGGTG-3'.

### 2.4. Western Blotting

Total proteins from cells and tissues were lysed in RIPA buffer (Beyotime Biotechnology, China) containing protease and phosphatase inhibitor Cocktail (MedChemExpress, China). Proteins were separated by 10% sodium dodecyl sulfate polyacrylamide gel (SDS-PAGE) and then transferred onto polyvinylidene difluoride membranes. Subsequently, the membranes were blocked with 5% nonfat milk for 1 h at room temperature and then incubated with primary antibodies and horseradish peroxidase (HRP)-conjugated secondary antibodies. The protein expression was finally visualized using an ECL kit (Invitrogen, USA). The following specific primary antibodies were used: anti-ASPM (Proteintech, USA), anti-cyclin E (Abcam, USA), anti-P21 (Cell Signaling Technology), anti-E-Cadherin (Proteintech), anti-N-cadherin (Proteintech), anti-Vimentin (Abcam), anti-*β*-catenin (Abcam), and anti-GAPDH (Proteintech).

### 2.5. Cell Transfection

ASPM shRNA oligonucleotide (clone TRCN0000118905) or negative control shRNA were cloned into pLKO.1 puro lentivirus vector (MISSION shRNA lentiviruses; Sigma‐Aldrich, St Louis, MO, USA) according to the manufacturer's instruction. The targeted ASPM shRNA sequence was 5′-CCGGCCAAAGTTGTTGACCGTATTTCTCGAGAAATACGGTCAACAACTTTGGTTTTTG-3'. The ASPM shRNA or negative control vectors were introduced into ATC cell lines using Lipofectamine 3000 (Invitrogen, USA). Cells overexpressing *β*-catenin were generated by transfection with *β*-catenin plasmids (human *β*-catenin pcDNA3; Addgene #16828). Gene manipulation efficiency was verified by qRT-PCR and western blot.

### 2.6. Cell Proliferation and Cell Cycle Analysis

Cell proliferation ability was measured by cell counting kit‐8 (CCK‐8; Beyotime Institute of Biotechnology) and colony information assay. For CCK-8 assay, cells were plated in 96-well plates, and then 10 ul CCK-8 solution was added to each well at indicated time points. Absorbance was determined at the wavelength of 450 nm. For colony information assay, cells were seeded in 6-well plates and grown for 14 days. Then, the cells were fixed with paraformaldehyde, followed by staining with 0.1% crystal violet solution, and cell colonies were counted and photographed.

For cell cycle distribution analysis, cells were washed with cold PBS and then fixed with 70% ethanol at 4 °C overnight, followed by staining with PI/RNase Staining Buffer (BD Biosciences, USA) for 30 min at 37°C. Subsequently, flow cytometry was applied using a flow cytometer (Becton Dickinson, USA). The number of cells in different phases of cell cycle was counted.

### 2.7. Cell Migration and Invasion Assays

For measuring migration capability, cells were placed in the upper chambers of the Transwell chambers (Corning Inc., USA) under a serum-free condition. For determining invasion ability, the cells were added to the top chambers precoated with Matrigel (BD Biosciences, USA) under a serum-free condition. DMEM containing 10% FBS was added to the bottom of the chambers. After incubation, the cells were fixed in 4% paraformaldehyde and stained with 0.1% crystal violet. Photos were taken using a digital microscope in random fields for each chamber.

### 2.8. Luciferase Reporter Assay

8505C and TTA1 cells were seeded in 24-well plates 24 h before transfection. The Wnt signaling reporter (TOPFlash or FOPFlash; Promega, USA) was co-transfected into 8505C and TTA1 cells along with lentivirus-mediated ASPM or control shRNA using Lipofectamine 3000 (Invitrogen) according to the manufacturer's protocol. The pRL-TK Renilla vector (Promega) was transfected as an internal control. Luciferase activity was measured with the Dual-Luciferase Reporter Assay Kit (Promega) 48 h following transfection. The data were normalized against Renilla luciferase activity and represented as TOPFlash/FOPFlash values.

### 2.9. Xenograft Model

Male Balb/c nude mice (6–8 weeks) from Beijing Huafukang Laboratory Animal Technology Co., Ltd. (Beijing, China) were used to generate xenograft models. All mice were kept for one week under specific pathogen-free conditions at room temperature (25 °C) with 12-h light/dark cycles for climatization before experiments. All animal procedures were carried out with the approval of the Animal Management and Use Committee of Tongji Medical College, Huazhong University of Science and Technology.

8505C cells were transfected with control or ASPM shRNA lentivirus vector to achieve sustained ASPM knockdown. Cells in 0.2 ml phosphate buffer saline (PBS)/Matrigel were subcutaneously implanted into athymic nude mice. Tumor volume was monitored every 3 days as well as the body weight of the mice. Tumor volume was calculated with the formula: volume = 1/2 × length× (width) [2].

### 2.10. Immunohistochemical (IHC) and Hematoxylin and Eosin (H&E) Staining

IHC analysis was performed according to the procedure as previously described [13]. Briefly, tumors were removed from the mice, fixed in 4% paraformaldehyde, embedded in paraffin, and serially sectioned at 4 µm thickness. After dewaxing, dehydration, and antigen repair, the sample sections were incubated with anti-ASPM antibody (Proteintech) or anti-Ki67 antibody (Abcam) at 4 °C overnight, followed by treatment with biotin-labeled secondary antibody (Proteintech). Tumor tissue sections were also stained with H&E for morphological examination. Tissue sections were visualized under a light microscope (Olympus, Japan).

### 2.11. Statistical Analysis

All data were analyzed with SPSS 22.0 statistical package software and expressed as mean ± standard deviation (SD). Unpaired independent Student's *t*-test was used for statistical comparisons between different groups. *P* < 0.05 indicated a significant difference.

## 3. Results

### 3.1. ASPM Is Upregulated in Human Samples and Cell Lines of ATC

**Figure 1:**
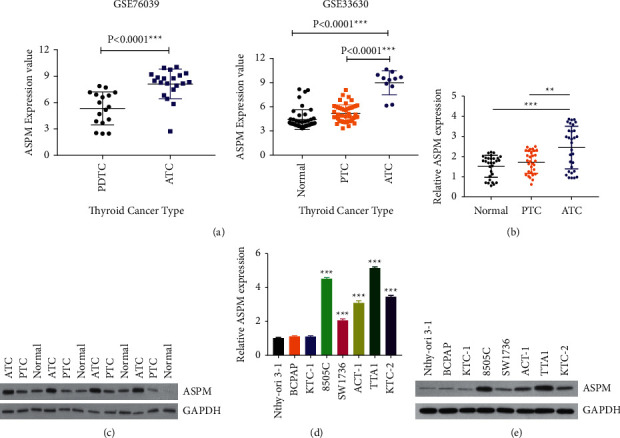
ASPM is upregulated in ATC patients and cell lines. (a) The expression of ASPM in ATC patients based on the data extracted from data sets GSE76039 and GSE33630. (b) The mRNA levels of ASPM in ATC (anaplastic thyroid cancer), PTC (papillary thyroid cancer), and normal thyroid tissues from enrolled patients. (c) The protein levels of ASPM in ATC, PTC, and normal thyroid tissues from enrolled patients. (d) The mRNA levels of ASPM in ATC cell lines. (e) The protein levels of ASPM in ATC cell lines. ^*∗∗*^*P* < 0.01, ^*∗∗∗*^*P* < 0.001.

Through analysis of mRNA expression data from microarray data sets GSE76039 and GSE33630, we found that ASPM levels were dramatically upregulated in human ATC tissues compared with that in PDTC tissues (poorly differentiated thyroid cancer), PTC tissues (papillary thyroid cancer), and normal tissues (Figure 1(a)). To further profile the expression pattern of ASPM in ATC tissues, we examined ASPM mRNA and protein expression in ATC, PTC, and normal thyroid tissues (*n* = 30 per group). In accordance with the bioinformatics analysis, the mRNA and protein levels of ASPM were both significantly elevated in ATC tissues relative to PTC and normal tissues (Figures 1(b) and 1(c)). Subsequently, we ulteriorly identified the same expression profile of ASPM in several human ATC cell lines (SW1736, 8505C, TTA1, C643, and ACT-1) via qRT-PCR (Figure 1(d)) and western blotting (Figure 1(e)). Compared with human PTC cell lines (BCPAP and KTC-1) and the normal thyroid epithelial cell line Nthy-ori 3-1, ASPM levels were higher in all the five ATC cell lines, especially in 8505C and TTA1 cells.

### 3.2. ASPM Depletion Inhibits the Malignant Properties of ATC Cells

**Figure 2:**
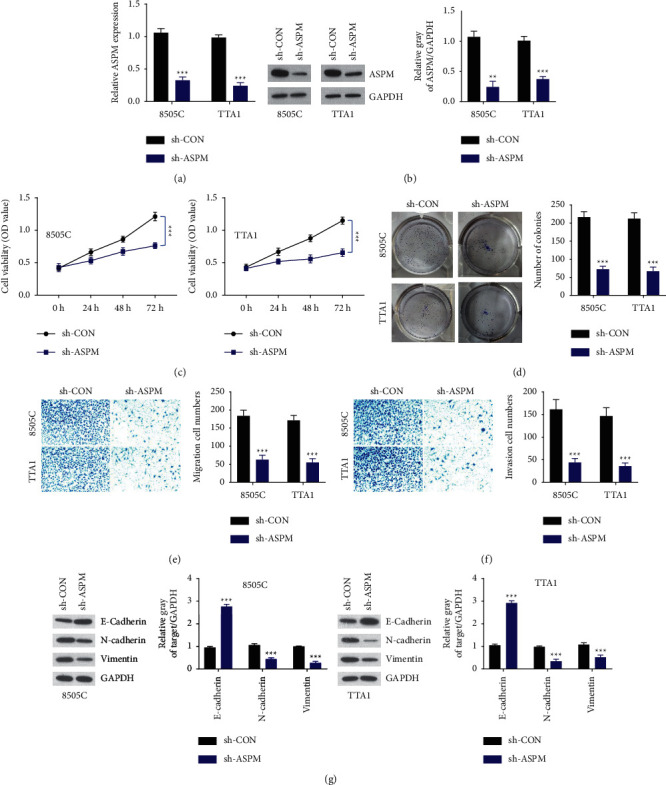
ASPM depletion inhibits proliferation, migration, and invasion of ATC cells. ((a), (b)) Validation of ASPM knockdown in 8505C and TTA1 cells by qRT-PCR and western blotting. ((c), (d)) ATC cell proliferation was evaluated by CCK-8 assay and colony formation assay. ((e), (f)) Migration and invasion abilities of ATC cells were assessed by Transwell assay. (g) The protein levels of E-cadherin, N-cadherin, and vimentin in ATC cells after transfection of sh-ASPM. ^*∗∗*^*P* < 0.01, ^*∗∗∗*^*P* < 0.001.

Given the overexpression of ASPM in ATC tissues and cells, we hypothesized that it played a pathogenic role in ATC progression. To address this, ASPM shRNA was introduced into 8505C and TTA1 cells for their highest expression of ASPM among the five ATC cell lines to stably knockdown the expression of ASPM in vitro. As shown in Figures 2(a) and 2(b), the transfection effectively suppressed ASPM expression at the mRNA and protein level in both 8505C and TTA1 cells. Since aberrant cell proliferation contributes to the deterioration of tumors, proliferation capacities of ASPM-knockdown cells were measured. A remarkable decline of the absorbance value was found in 8505C and TTA1 cells after ASPM depletion, as illustrated by CCK-8 assays (Figure 2(c)). Analogously, we also found that cell growth was blocked by ASPM knockdown through colony formation assays (Figure 2(d)). In addition, Transwell assays were carried out to investigate the effects of ASPM on migration and invasion abilities of ATC cells. As expected, following ASPM knockdown, an observable inhibition of migration and invasion properties was noticed in both 8505C and TTA1 cells (Figures 2(e) and 2(f)). For further validation, we also demonstrated that E-cadherin expression was increased while the levels of epithelial mesenchymal transition (EMT)-related proteins (N-cadherin, vimentin) decreased upon in vitro ASPM knockdown (Figure 2(g)). These results collectively indicate that ASPM could function as an oncogenic factor in ATC progression.

### 3.3. ASPM Abrogation Induces Cell Cycle Arrest

**Figure 3:**
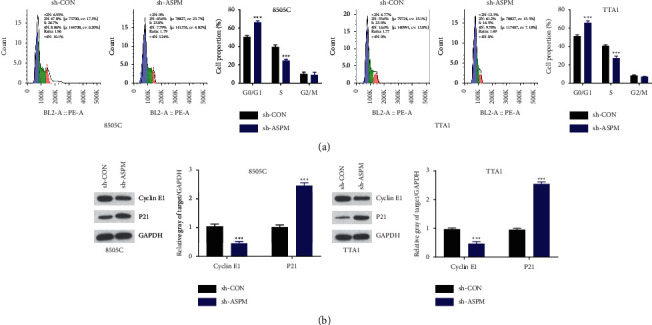
ASPM inhibition induces cell cycle arrest of ATC cells. (a) Cell cycle was analyzed by flow cytometry. (b) The expression of cyclin E1 and P21 was detected using western blotting. ^*∗∗∗*^*P* < 0.001.

To further explore whether cell cycle arrest is involved in the ASPM-mediated regulation of ATC cell growth, PI staining was employed to identify the effect of ASPM abrogation on cell cycle. It has been shown that ASPM contributes to cyclin E abundance through G1 restriction point [7]. In our study, cell cycle analysis showed that ASPM depletion led to arrest at the G0/G1 phase in 8505C and TTA1 cells, characterized by increased proportion of cells in the G0/G1 phase and decreased proportion in the S phase (Figure 3(a)). Additionally, we also observed that downregulation of ASPM reduced cyclin E and p21 levels (Figure 3(b)). Taken together, ASPM suppression could induce cell cycle arrest of ATC cell lines via cyclin E.

### 3.4. Wnt/*β*-Catenin Signaling Is Blocked by ASPM Knockdown

**Figure 4:**
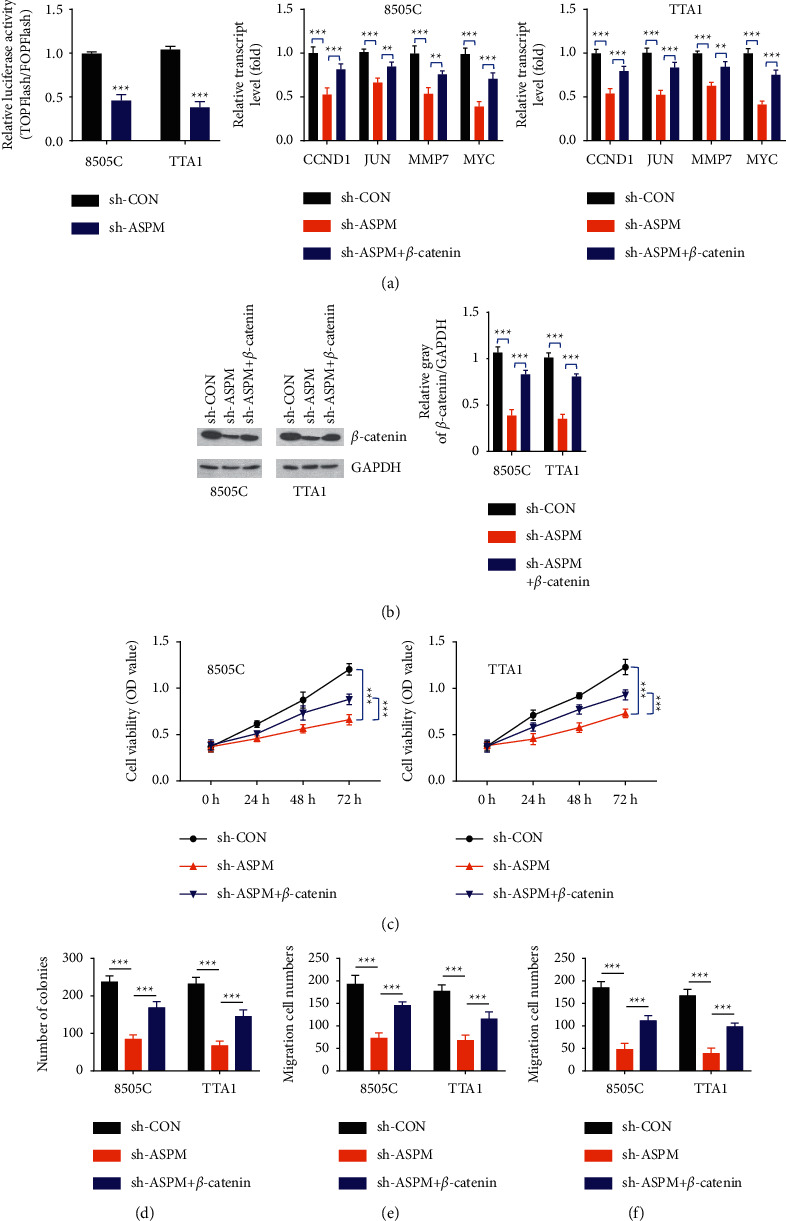
ASPM regulates the Wnt/*β*-catenin signaling pathway in ATC cells. (a) Wnt3a was detected by TOPFlash assay, and several representative targets of Wnt signaling (CCND1, c-Jun, MMP7, and c-MYC) were evaluated using western blotting. (b) The expression of *β*-catenin in ATC cells after co-transfection with sh-ASPM and human *β*-catenin plasmids. (c–f) Cell proliferation, migration, and invasion were re-evaluated after the rescue of *β*-catenin. ^*∗∗*^*P* < 0.01, ^*∗∗∗*^*P* < 0.001.

As reported previously, ASPM is identified as a novel regulator of Wnt signaling, facilitating neurogenesis in the developing brain and contributing to the oncogenicity in cancer cells [9,11,12]. We, therefore, assumed that the Wnt/*β*-catenin pathway is also involved in ASPM-mediated malignant behaviors of ATC cells. To confirm our hypothesis, TOP/FOPFlash assay was employed. As expected, the luciferase activity in ASPM-knockdown cells was obviously lower in contrast to that in control cells (Figure 4(a)). Simultaneously, the levels of several representative targets of Wnt signaling, including CCND1, c-Jun, MMP7, and c-MYC, were reduced upon downregulation of ASPM in ATC cells (Figure 4(a)). Afterwards, 8505C and TTA1 cells were co-transfected with ASPM shRNA and human *β*-catenin plasmids to restore *β*-catenin expression. 48 h after transfection, the expression of *β*-catenin was rescued as shown in Figure 4(b). Following the rescue of *β*-catenin in ATC cells, the reduction of Wnt signaling target genes (Figure 4(a)) and the impaired proliferation abilities of cancer cells induced by ASPM knockdown were obviously restored (Figures 4(c) and 4(d)). Similarly, cell migration and invasion were also improved upon *β*-catenin rescue, both in 8505C and TTA1 cells (Figures 4(e) and 4(f)). Collectively, these findings reveal that ASPM regulates the malignant properties of ATC cells through the Wnt/*β*-catenin signaling pathway.

### 3.5. ASPM Promotes Tumorigenesis of ATC In Vivo

**Figure 5:**
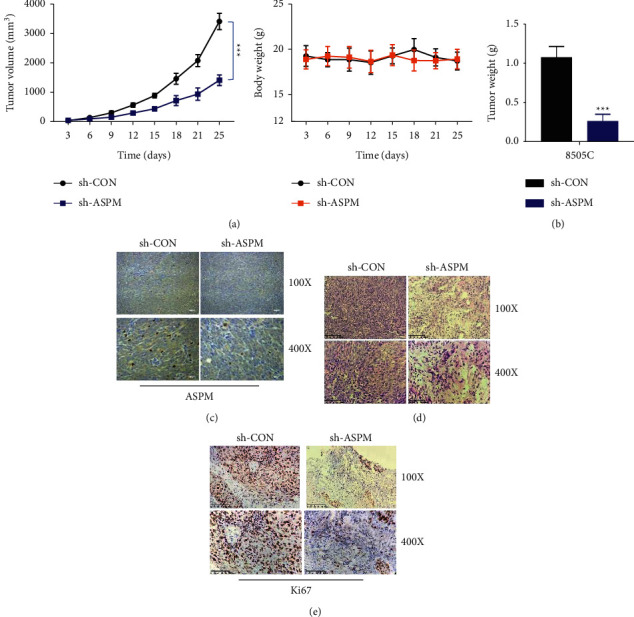
ASPM promotes subcutaneous tumor growth in nude mice. (a) Tumor volume and mice body weight. (b) Tumor weight and gross morphology. (c) ASPM expression was evaluated by immunohistochemistry. (d) H&E staining of tumor tissues. (e) Ki67 staining in tumor tissues. ^*∗∗∗*^*P* < 0.001.

Given the oncogenic role of ASPM in vitro, we next sought to explore its tumorigenicity in a xenograft tumor model. Pretransfected 8505C cells were subcutaneously inoculated into athymic nude mice to determine the xenograft tumor growth in vivo. The volume and weight of the xenograft tumors were compared 25 days after implantation. As shown in Figures 5(a) and 5(b), compared with mice treated with control cells, the volume and weight of the subcutaneous tumors in mice treated with ASPM-knockdown cells were dramatically smaller. The IHC analysis of tumor tissues showed a significant decrease of ASPM expression in the ASPM-knockdown group (Figure 5(c)). Histological analysis was also carried out by H&E staining (Figure 5(d)). Ki67 staining also revealed attenuated cell proliferation in tumor tissues from the sh-ASPM group (Figure 5(e)). Taken together, these results indicate a tumorigenic role of ASPM in vivo.

## 4. Discussion

Increasing studies have shown that ASPM is overexpressed in multiple tumor tissues and is associated with disease progression [5,17]. ASPM was highly expressed in the hepatocellular carcinomas and was correlated with metastatic potential and tumor recurrence [18]. Aberrant expression of ASPM facilitated the tumorigenesis of malignant gliomas [19–21]. In addition, an association between ASPM and poor clinical features was also identified in epithelial ovarian cancer, and ASPM was involved in cancer progression [22]. In the present study, for the first time, we found that ASPM mRNA expression was upregulated in human ATC tissues through bioinformatics analysis and further confirmed the results in human samples collected from our hospital and ATC cell lines. These results indicate that ASPM overexpression may be implicated in the progression of ATC.

As demonstrated in several studies, ASPM contributes cell proliferation and invasive properties of different cancer cells, including prostate cancer [11], lung squamous cell carcinoma [13], glioblastoma [17], pancreatic cancer [10], and bladder cancer [23]. High expression of ASPM in ATC suggests an involvement of ASPM in the development of ATC. To address this possibility, ASPM expression in ATC cells was downregulated by gene manipulation. Similar to the previous findings, we also found that ASPM depletion inhibited the proliferation, migration, and invasion of ATC cells. In addition, our study also revealed that silencing ASPM in vitro resulted in an increase of the epithelial cell adhesion molecule, E-cadherin, and decreased EMT markers (N-cadherin, vimentin). EMT activation leads to a transition of epithelial cells into invasive mesenchymal cells, contributing to the metastatic process of cancer cells [24]. It is known that suppression of E-cadherin enhances cancer metastasis [25]. It is also reported that E-cadherin is beneficial to inhibit ATC cell invasion [26]. As essential EMT-associated factors, vimentin and N-cadherin are indispensable regulators in mesenchymal cell migration and are involved in the metastasis of ATC [24].

As reported previously, ASPM regulates the orientation of spindles in the metaphase of mitosis, and the abrogation of ASPM could result in cell cycle disorder [27]. Besides, ASPM could stabilize cyclin E through G1 restriction point, thereby regulating mitosis duration [7]. Here in this study, we verified that knockdown of ASPM reduced cyclin E expression and induced an arrest at the G0/G1 phase in ATC cells, through which cell proliferation was significantly inhibited. The results are consistent with earlier reports where ASPM facilitates glioblastoma cell growth via regulating G1 restriction point [17].

Our data showed that Wnt/*β*-catenin signaling activity was suppressed upon ASPM knockdown, and rescue of *β*-catenin expression restored the proliferation, migration, and invasion properties of ATC cells, implying the involvement of Wnt/*β*-catenin pathway in the progression of ATC. As we know, *β*-catenin is a crucial mediator of Wnt signaling [28]. Cumulative studies have emphasized the critical role of Wnt/*β*-catenin signaling in thyroid cancer, including ATC [29]. Besides, there is compelling evidence supporting the role of ASPM in the activation of Wnt/*β*-catenin pathway [9,12]. Prior studies have also identified that ASPM-mediated activation of Wnt/*β*-catenin signaling contributes to the tumorigenicity of cancer cells [11,17], which is in line with our findings. Finally, the xenograft model further validated the oncogenic role of ASPM in vivo.

The undifferentiated and aggressive phenotype of ATC contributes to its dismal prognosis [30]. Since loads of patients with ATC inevitably presented with metastasis upon initial diagnosis, the curative effect of current therapeutic options is limited for ATC [31]. Hence, it is of great priority to identify optimal treatment targets of ATC. Herein, we proposed that ASPM, a cell centrosome protein, is overexpressed in ATC tissues and acts as a tumorigenic factor in vivo and in vitro, thus making it a promising novel target for ATC treatment.

## Data Availability

The data generated and analyzed during the current study are available from the corresponding author on reasonable request.
